# Dyke-Davidoff-Masson-like syndrome in an adult cat

**DOI:** 10.1177/20551169241273691

**Published:** 2024-09-10

**Authors:** Andrea Thon, Elisa Gamperl-Mikula, Florian Willmitzer, Michael Leschnik, Kristina Anna Lederer

**Affiliations:** 1 Tierklinik Parndorf, VHB – Veterinaria Health Betriebs GmbH, Parndorf, Austria; 2 Kappa1 – Veterinary Radiology Services, Zurich, Switzerland; 3 Department for Companion Animals and Horses, Internal Medicine, University of Veterinary Medicine, Vienna, Austria; 4 Department for Companion Animals and Horses, Diagnostic Imaging, University of Veterinary Medicine, Vienna, Austria

**Keywords:** Dyke-Davidoff-Masson-like syndrome, cerebral hemiatrophy, cerebral hypoplasia, calvarial hyperostosis, cerebral hypoperfusion, seizures, neurology

## Abstract

**Case summary:**

A 4-year-old cat was presented with acute onset of lateralised neurological central nervous system (CNS) signs and seizures. Haematological and serum biochemical parameters were within normal limits. Imaging diagnostics revealed severe CT and MRI abnormalities of the right brain, similar to Dyke-Davidoff-Masson syndrome (DDMS) in human medicine. This syndrome includes cerebral hemiatrophy with compensatory calvarial hyperostosis and ventriculomegaly. Such changes have previously been reported only once in a single feline case of approximately the same age. In humans, DDMS is described as an embryonic and perinatal developmental disturbance or an acquired injury in early childhood.

**Relevance and novel information:**

This case report shows that without further imaging diagnostics, congenital disorders can be overlooked in some rare cases of adult cats with later onset of their first clinical signs.

## Case description

**Figure 1:**
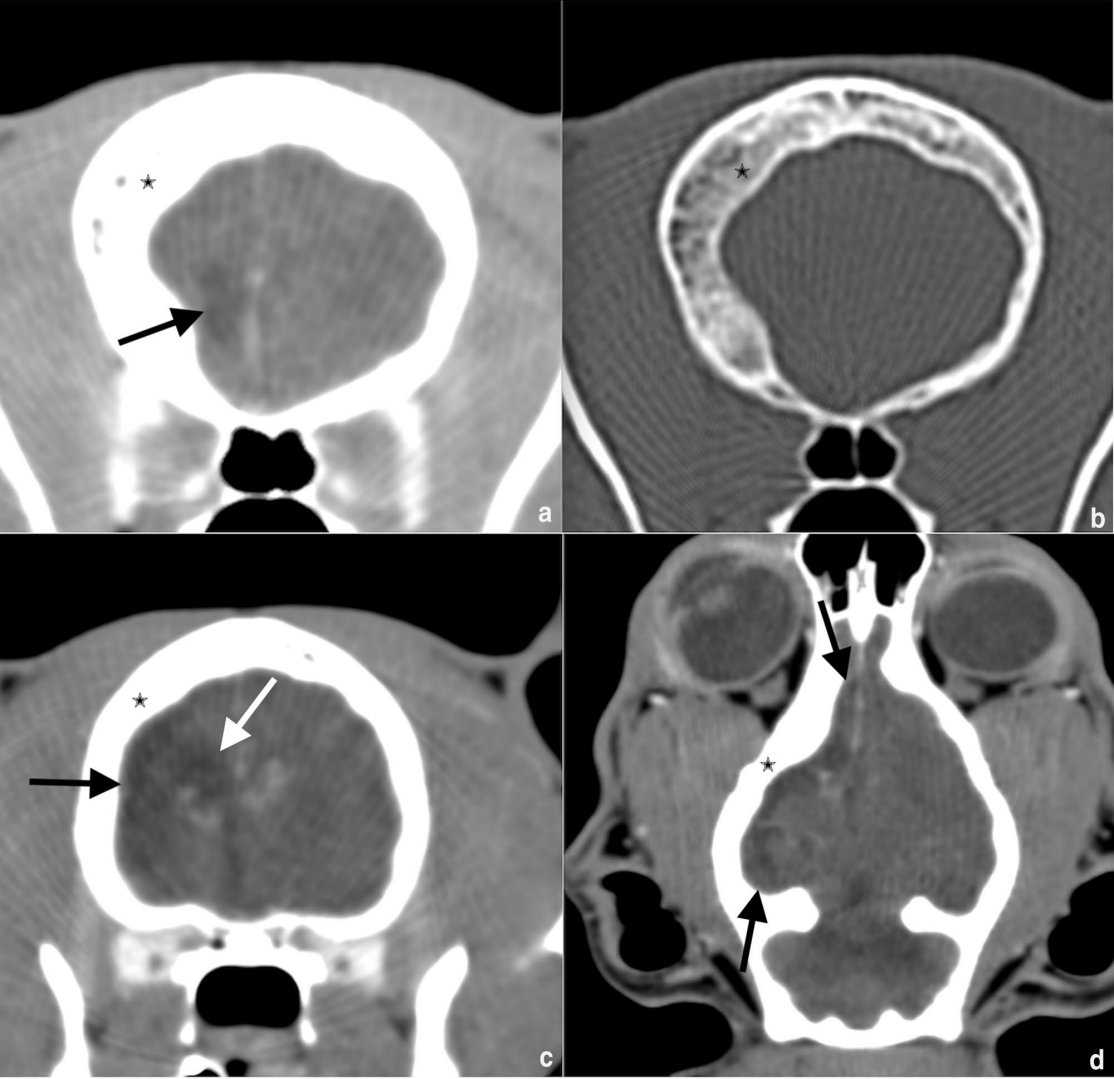
Comparative transversal images in (a) soft tissue algorithm before contrast, (b) bone algorithm before contrast, and (c,d) transversal and dorsal reconstructed images in soft tissue algorithm after contrast (the patient’s right is to the left of the image). Note the asymmetry of the calvarium and severe expansion of the diploic space (asterisk). Severe reduction of the volume of the right cranial cavity and of the right forebrain compared with the left side. A large part of the right-sided frontal and parietal lobes was fluid-like attenuating (black arrow), with suspicion of direct communication to a severe dilated right lateral ventricle (white arrow)

**Figure 2:**
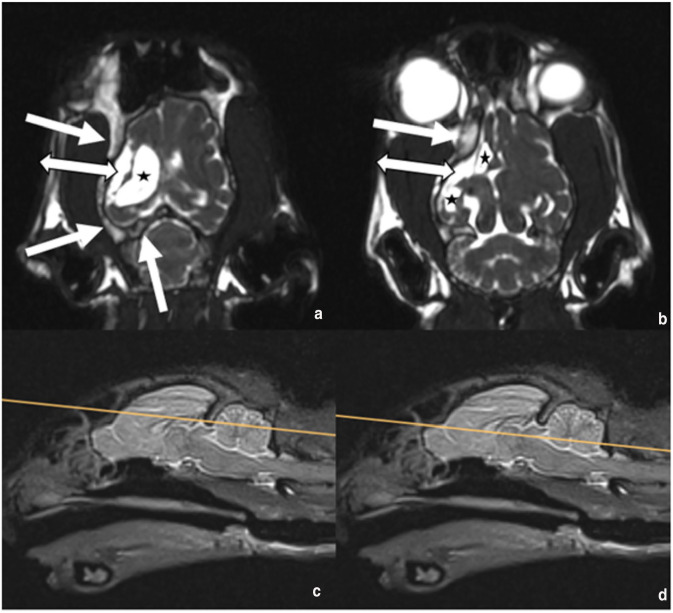
(a,b) Dorsal CISS and (c,d) sagittal T2W images. (c, d) The sagittal T2W images are reference images for the dorsal planes (a,b), respectively. (a,b) There is right cerebral hypoplasia with ipsilateral calvarial thickening with widening of the diploic space (white arrow), the subarachnoid space is distended (double-headed arrow) and there is dilatation of the right lateral ventricle (asterisk). For all transverse images, the patient’s right is to the left of the image. For sagittal images, rostral is to the left. CISS = constructive interference in steady state; T1W = T1-weighted; T2W = T2-weighted

**Figure 3:**
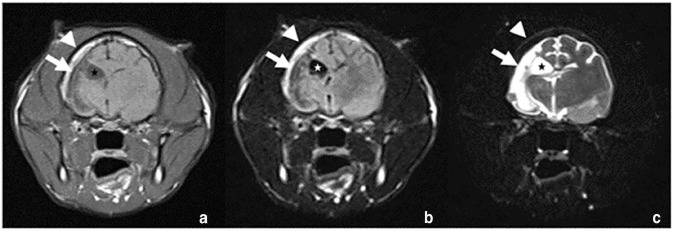
(a) Transverse T1W, (b) T2W FLAIR and (c) T2W FS images. Note the midline shift towards the right due to hypoplasia of the right forebrain. The right lateral ventricle is moderately distended (asterisk). Distension of the right subarachnoid space (arrow). (b) There is complete suppression of the CSF signal of the lateral ventricle and moderate suppression of the CSF signal in the subarachnoid space. (c) T1 and T2 FLAIR hyperintense signal of the thickened calvarium (arrowhead) with signal suppression in the T2W FS image. For all images, the patient’s right is to the left of the image. CSF = cerebrospinal fluid; FLAIR = fluid-attenuated inversion recovery; FS = fat-saturated; T1W = T1-weighted; T2W = T2-weighted

A 4-year-old male castrated domestic shorthair cat was presented with an acute onset of ataxia, clockwise circling, rotatory nystagmus and vomiting. The cat had been owned for 2 years and originated from Romania. It had regular outdoor access, received two vaccinations 6 months previously and was dewormed regularly. The cat weighed 4.5 kg and was fed a commercial diet. It had always seemed clumsy to the owners.The cat initially showed a progressive course of neurological signs for several hours, followed by spontaneous stabilisation. The cat was presented with tight circling to the right, right-side accentuated ataxia and truncal swaying. In addition, a lack of menace response in the left eye as well as a normal blinking reflex pointed to a central nervous system (CNS) lesion. Pupillary light reflex was normal. An increased extensor tone on the left legs as well as decreased extensor tone on the right legs were noted. The postural reaction was mildly delayed in both left limbs and the spinal reflexes were unremarkable. A vestibular/cerebellar localised lesion was suspected. A differential diagnosis included neoplasia and haemorrhage.A complete blood count (CBC), blood chemistry and a feline immunodeficiency virus (FIV)/feline leukaemia virus (FeLV) test (SNAP; IDEXX) were unremarkable. The cat was hospitalised for further examinations and treated with prednisolone 1.5 mg/kg once, followed by 1 mg/kg q12h, pantoprazole (0.5 mg/kg q12h), maropitant (1 mg/kg q24h) and intravenous constant rate infusion.A CT scan including contrast study and cerebrospinal fluid (CSF) sampling were performed 2 days after the first presentation. The cat underwent a CT examination of the head under general anaesthesia using a 16-slice CT scanner (Activion 16; Toshiba), images were acquired in helical mode and reconstructed in soft tissue and bone algorithms with a slice thickness of 0.5 mm. After the native scan, a 600-iodine mg/kg intravenous bolus of lobiriol (Xenetix 300 mg/ml) was administered, imaging was repeated after a delay of 90 s. In the bone algorithm, the outer table of the calvarium was symmetrical. There was a severe thickening of the inner table of cortical bone and expansion of the diploic space in the right part of the calvarium. The right frontal sinus had greater volume than the left and was extended more caudally in the rostral part of the thickened diploic space. The rest of the diploic space was filled with tissue-like cancellous bone. Secondarily, there was a volume reduction of the right cranial cavity and the right forebrain compared with the left side. A large part of the right frontal and parietal lobes was fluid-like attenuating, with suspicion of direct communication to a severe dilated right lateral ventricle, which was interpreted as loss or agenesis of brain parenchymal tissue replaced by CSF. In addition, a midline shift to the right was present in the rostral part of the cerebrum (Figure 1). Altogether, the findings were compatible with Dyke-Davidoff-Masson-like syndrome. CSF was collected from the cerebellomedullary cistern and revealed a differential cellular count of mononuclear cells (approx. 1:1 small lymphocytes and macrophages) and single neutrophil granulocytes indicating a mild mononuclear pleocytosis.Four weeks after the original presentation, the cat had a 10-min episode of orofacial automatism. At that time, circling and pleurothotonus to the left and a left-sided proprioception deficit were documented. Phenobarbitone 2.8 mg/kg q12h PO was started.Seven weeks after the original presentation, MRI was performed. The patient was routinely anaesthetised, and MRI of the head was performed using a 1.5 T scanner using a human knee coil (Magnetom Avanto; Siemens). After premedication with butorphanol, the cat had a short, self-limiting generalised epileptic episode. Transverse images were obtained with T2-weighted (repetition time [TR] 5370, echo time [TE] 111, slice thickness 2.5 mm), T2 fluid-attenuating inversion recovery (FLAIR; TR 8500, TE 86, slice thickness 3 mm), T2 fat-saturated (FS; TR 5890, TE 111, slice thickness 5 mm), constructive interference in steady state (CISS) (TR 6.32, TE 2.72, slice thickness 1.5 mm), T2* (TR 800, TE 26, slice thickness 2 mm), diffusion-weighted imaging (DWI; TR 3800, TE 113, slice thickness 2.8 mm) with a corresponding apparent diffusion coefficient (ADC) map, T1-weighted (TR 907, TE 14, slice thickness 2.5 mm) sequences before and after administration of the intravenous gadolinium-based contrast agent (gadoterate meglumine; Dotarem). Sagittal 3D images were obtained with T2-weighted (TR 3000, TE 388, slice thickness 0.8 mm) and T1-weighted images (TR 1720, TE 5.53, slice thickness 0.9 mm) before and after contrast administration. Dorsal plane CISS 3D sequences were acquired (TR 6.33, TE 2.72, slice thickness 1 mm). The right cerebral hemisphere, as well as the right diencephalon and mesencephalon, were markedly decreased in volume with secondary enlargement of the subarachnoid space and dilatation of the right lateral ventricle (Figures 2 and 3). The ipsilateral calvarium (frontal bone, squamous part of temporal bone, parietal bone and tentorium cerebelli osseum) was moderately thickened with widening of the diploic space (Figure 2). Because of the hypoplasia of the right cerebral hemisphere, there was a midline shift towards the right (Figure 3). No pathological signal intensities were identified after administration of the intravenous contrast agent. The cerebellum and craniocervical junction were within normal limits.

The cat was discharged to home care with prednisolone 1 mg/kg q24h and phenobarbitone 2.8 mg/kg q12h. One-and-a-half years later, the cat was reported to be doing well, was without seizures and showing no side effects of ongoing treatment. Haematology and blood chemistry were unremarkable, and the cat showed no clinical signs other than a mild anisocoria (right pupil > left pupil).

## Discussion

Cerebral hemiatrophy caused by various congenital or acquired diseases was first described in human medicine by Rondão et al^1^ and has since been described as Dyke-Davidoff-Masson syndrome (DDMS). It is caused by brain injury leading to hypoplasia of one cerebral hemisphere. The congenital form is characterised by cerebral injury that occurs in the intrauterine or neonatal period, often due to vascular aetiologies.^2^ A common pathophysiological hypothesis postulates cerebral hypoperfusion causes venous congestion and a reduction of neurotrophin levels leading to unilateral underdevelopment. Clinical signs are present at birth or a short time thereafter and include epileptic seizures, contralateral hemiparesis, craniofacial asymmetry, unstable gait, cognitive dysfunction and sensory disorders.^1^ The most important acquired causes include trauma, infection, vascular anomalies, ischaemic and haemorrhagic conditions, and subependymal germinal matrix and intraventricular haemorrhages in premature infants or at any time thereafter. Imaging examinations demonstrate unilateral cerebral hypoplasia, calvarial thickening, dilated sulci and a falx cerebri shift.^1^ A definitive pathological differentiation between primary and secondary causes is often not possible.^3^ Two authors of a small case series in human medicine suggested that widened sulci are present if the vascular insult occurs after birth or after sulcation is complete. If the vascular insult occurs during embryogenesis (before formation of gyri and sulci is completed), prominent sulci will not be present.^2,3^ However, in another case series, not all acquired cases of cerebral hemiatrophy had widened sulci.^4^ The morphological aspects of embryonic and fetal development in cats are similar to the development of the human nervous system;^5^ therefore, embryonic and perinatal developmental disturbance as well as acquired injury may lead to comparable cerebral as well as skull malformation.^6,7^ The CNS in cats develops until complete maturation is reached within 12–18 months.^8^ The cerebrocortical surface in cats expands to nearly 90% of adult values between the third and sixth weeks of life.^9^ In cats, only one case report describes a DDM-like syndrome with similar changes on CT and MRI. The adult cat presented with seizures and delayed postural reactions in the left thoracic and pelvic limbs. On MRI, portions of the right frontal, parietal and temporal lobes were absent and changes in the right frontal and parietal bones were characterised by a thickening of the inner and outer table of cortical bone and expansion of the diploic space.^10^

Our case report describes an adult cat with an acute onset of lateralised clinical signs and the findings in the CT and MRI scans are very similar to those in human DDSM and in one published feline case report.^1,10^ The cat presented with suspicious changes, especially those in the cranium, due to an early loss of parenchyma. There was no widening of sulci, more likely indicating a congenital cause than an acquired injury. The acute onset of clinical signs remains ambiguous as all documented morphological changes were classified as stable with presumed development during the perinatal period. Additional pathological conditions might be assumed as a trigger for the acute appearance of clinical signs, also supported by the stable condition 1.5 years later. Potential comorbidities, such as brain inflammation, cerebrovascular disease or increased intracranial pressure, are possible explanations for acute clinical signs. Patients with intracranial malformations might be more susceptible to such additional brain-related morbidities, and an age-related reduction in brain mass could also aggravate these conditions. The lateralised clinical signs in this cat can be explained by the cerebral asymmetric changes and are comparable to DDMS in humans.^1^ Epileptic seizures are the main reason for diagnostic work-up; therefore, the mean age at diagnosis of human DDMS is 14–23 years, which applies to both cats described, and corresponds to a young adult stage.^1,10,11^ In the congenital variant of DDMS, additional clinical signs, such as hemiparesis or clumsy motion patterns, are usually recognised, as also described in this feline case.^10,11^ A difference in DDMS in human medicine might be the frequently described craniofacial asymmetry. This morphology was not observed in cats and could be related to the different vascular supply of the head and facial region in cats and humans.^2,6,10,12^ This cat and the one previously described showed alterations of the right hemisphere, whereas in humans, changes of the left hemisphere are overrepresented (69%).^13^ Possible differential diagnoses were porencephaly and hydrocephalus ex-vacuo with concurrent calvarial hyperostosis. The latter has been excluded considering the midline shift to the right, which indicates loss of volume of the right cerebral hemisphere rather than a mass effect. Moreover, no peripheral space-occupying lesion and no contrast enhancement were present, indicating a cystic meningioma. The asymmetry in morphology of the frontal bone and the corresponding sinus cannot be explained by hydrocephalus or porencephaly.^7^

## Conclusions

DDMS is a rare condition in human medicine and also seems to be a seldom diagnosis in feline medicine. This case report describes the CT and MR findings of cerebral hemiatrophy and should help to identify this uncommon entity. Each additional case improves the understanding of this feline neurological condition and allows more precise comparability with the human syndrome.

## References

[1] Rondão MBA Hsu BRRHS Centeno RS , et al. Dyke-Davidoff-Masson syndrome: main clinical and radiological findings- systematic literature review. Seizure 2023; 110: 58–68. 37327751 10.1016/j.seizure.2023.04.020

[2] Anguiar PH Lui CW Leitao H , et al. MR and CT imaging in the Dyke-Davidoff-Masson syndrome: report of three cases and contribution to pathogenesis and differential diagnosis. Arq Neuropsiquiatr 1998; 56: 803–807. 10029885 10.1590/s0004-282x1998000500016

[3] De Lahunta A Glas E . Development of the nervous system malformation. In: de Lahunta A Glass E (eds). Veterinary neuroanatomy and clinical neurology. St Louis, MO: Saunders 2009, pp 23–76.

[4] Zilkha A. CT of cerebral hemiatrophy. AJR Am J Roentgenol 1980; 135: 259–262. 6773323 10.2214/ajr.135.2.259

[5] De Morais-Pinto L da Veiga ML Almeida da Anunciação AR . Central nervous system development of cats (*Felis catus* L1758). Res Vet Sci 2021; 141: 81–94. 34700148 10.1016/j.rvsc.2021.10.015

[6] Sener RN Jinkins JR. MR of craniocerebral hemiatrophy. Clin Imaging 1992; 16: 93–97. 1547482 10.1016/0899-7071(92)90119-t

[7] MacKillop E. Magnetic resonance imaging of intracranial malformations in dogs and cats. Vet Radiol Ultrasound 2011; 52: S42–S51. 10.1111/j.1740-8261.2010.01784.x 21392155

[8] Noden DM de Lahunta A. Central nervous system and eye. In: Nodem DM de Lahunta A (eds). The embryology of domestic animals: developmental mechanisms and malformations. Baltimore, MD: Williams and Wikings, 1985, pp 92–119.

[9] Rathjen S Engelmann R Struif S , et al. The growth of cat cerebral cortex in postnatal life: a magnetic resonance imaging study. Eur J Neuosci 2003; 18: 1797–1806. 10.1046/j.1460-9568.2003.02909.x 14622214

[10] Song RB Glass EN Kent M , et al. Magnetic resonance imaging and computed tomography findings of Dyke-Davidoff-Masson-like syndrome in a cat. Aust Vet J 2015; 93: 377–380. 26412120 10.1111/avj.12365

[11] Shahid R. An unusual presentation of Dyke-Davidoff-Masson syndrome. Neurosciences (Riyadh) 2018; 23: 254–257. 30008003 10.17712/nsj.2018.3.20170232 PMC8015585

[12] Bagazgoitia L García-Penas JJ Duat-Rodríguez A , et al. Facial capillary malformation and Dyke-Davidoff-Masson syndrome. Pediatr Neurol 2010; 43: 202–204. 20691943 10.1016/j.pediatrneurol.2010.04.011

[13] Duncan MA Vazquez-Flores S Chávez-Lluévanos EB , et al. Dyke-Davidoff-Masson syndrome: a case study. Medicina Universitaria 2014; 16: 71–73.

